# Supplementary material for: Patterns of Intron Gain and Loss in Fungi
Source: PLoS Biol. 2004 Nov 30;2(12):e422. doi: 10.1371/journal.pbio.0020422 (PMC532390; doi:10.1371/journal.pbio.0020422)
Supplement: Table S1 — Also available at http://genes.mit.edu/NielsenEtAl/. (4.3 MB ZIP). [file pbio.0020422.st001.zip › NielsenEtAl/html/268.html]

AN1797.1.NCU04963.1.MG06203.1.FG02641.1


```
 CLUSTAL W (1.82) Multiple Sequence Alignments - Introns Inserted


Sequence 1: MG06203.1	535 aa
Sequence 2: FG02641.1	540 aa
Sequence 3: NCU04963.1	523 aa
Sequence 4: AN1797.1	512 aa
Alignment Length: 546 aa
Number Identitical Residues: 249 aa
Alignment Score (without introns) 12869


MG06203.1 	---MVRGFKRPENEAGAAWPAILVGAFVSFGGVLFG2YDTGTISGILAMKYWKELFSTGY
NCU04963.1	---MGLSLKKPEGVPGKSWPAIVIGLFVAFGGVLFG2YDTGTIGGILAMPYWQDLFSTGY
FG02641.1 	MNTLRRASLSKPEEAGKTWPAIAIGAFVAFGGVLYG2YDTGTISGILAMPYWQSLFSTGY
AN1797.1  	---MGFMLRKPDDAAGSAAPAILIGLFVAFGGVLYG2YDTGTISGILAMKYWRRQFSTGY
          	   :          .* : *** :* **:*****:* ******.***** **:  *****

MG06203.1 	TDATGQL-GISPSEEAAVVSILSAGTFFGALASVFLADSIGRRWALIASCWVFNLGVIFQ
NCU04963.1	RNPEHHL-DVTASQSATIVSILSAGTFFGALGAAPLADWAGRRLGLILSSFVFIFGVILQ
FG02641.1 	RDAKGHL-NITTAQESGIVSILSAGTFFGALSSPFMTDYIGRRPGLMIATWVFNLGVCLQ
AN1797.1  	VNPNDHIPDVTSSQSSMIVSLLSAGTFFGALTAAPVADYFGRRIAMILDCFVFCFGVVLQ
          	 :.  ::..::.::.: :**:********** :  ::*  *** .::   :** :** :*

MG06203.1 	TAAVALPLFLAGRFFAGFGVGLISAMI~PLYQSETAPKWIRGAIVGAYQLSITIGLLLAA
NCU04963.1	TAAVSIPLFLAGRFFAGLGVGLISATI1PLYQSETAPKWIRGVIVGSYQLAITIGLLLAS
FG02641.1 	VAATSIPLFLAGRFFAGFGVGQISAII1PLYQSETAPKWIRGAIVGSYQWAITIGLLLAA
AN1797.1  	TAATAIPLFVAGRFFAGFGVGLLSATV~PLYQSETAPKWIRGTIVGAYQLAITIGLLLAA
          	.**.::***:*******:*** :** : **************.***:** :********:

MG06203.1 	IVNNSTHERDDTGSYRIPIAVQFAWSIILIVGMLILPETPRFLVKKGKMDQAARSLGKLR
NCU04963.1	IVNNATHNMQNTGCYRIPIAVQFAWAIILIVGMIILPETPRFHIKRDNLPAATRSLAILR
FG02641.1 	IVNNATGGRNDTGSYRIPVAVQFAYSLILFGGMIILPETPRFLIKKDRHEAAAKALSRIR
AN1797.1  	IVNNSTKDRNDTGCYRIPVAVQFAWAIILVVGMIILPETPRFLIKQDNHEAAIKSLARLR
          	****:*   ::**.****:*****:::**. **:******** :*:..   * ::*. :*

MG06203.1 	RLPADHEAIVAEVAEIKANNDYEMSQGTAGYIDCFRGPML~KRQLTGMGLQALQQLTGVN
NCU04963.1	RLEQNHPAIIEELSEIQANHEFEKSLGKATYLDCLKGNLL~KRLLTGCFLQSLQQLTGIN
FG02641.1 	RLTPDHPAIQAELAEVRANHEYETSIGKSSYLDCFKPPIL~KRQFTGCALQALQQLTGIN
AN1797.1  	RLDINDLAVVEELEEIKANHEYELSVGKASYLEILRGSIV1T------------------
          	**  :. *:  *: *::**:::* * *.: *:: ::  :: .                  

MG06203.1 	FI~FYYGTKYFENSGISQPFVIQMITSVINVVSTIPGLIAVDRWGRRPMLLWGAIGMTIS
NCU04963.1	FI~FYYGTQFFKNSGFSDSFLISLITNLVNVVSTLPGLYAIDKWGRRPVLLWGAVGMCVC
FG02641.1 	FI0FYYGTKYFQNSGISSGFVISMITSAINVASTIPGMYAIDKWGRRPLLLWGAVGMCVS
AN1797.1  	--~VYYGTTFFQHSGINNSFVITLITNIVNVVSTLPGLWMVEKWGRRPLLLFGAVGMCVS
          	   .**** :*::**:.. *:* :**. :**.**:**:  :::*****:**:**:** :.

MG06203.1 	QFLVAMLGTLTTGQRADGSIIVYNVEAQKAGVAFVCIYIFFFASTWGPLAW~VVTGEIFP
NCU04963.1	QFIVAILGTTTTSQDASGMIIVHNLAAQKAAIAFICFYIFFFAASWGPVAW2VVTGEIFP
FG02641.1 	QFIVAMAGTFSTGQNADGTIFVKSLAGQKAAVSFVCIYIFFFASTWGPLAW~VVTGEIFP
AN1797.1  	QFIVAIVGTTTDSGVAN-----------KVLIAFVCVYIFFFASSWGPVAW~VVTGELFP
          	**:**: ** : .  *.           *. ::*:*.******::***:** *****:**

MG06203.1 	LKQRARGLSLTTATN0WLLNWAIA~YMTPYLVNFGPGYANLQSKIFFVWFGACFLCIAFV
NCU04963.1	LKVRAKSLSITTASN~WLLNWAIA~YSTPYLVNYGPGNANLQSKIFFVWGGCCFICIAFV
FG02641.1 	LKTRAKSLSMTTATN~WLFNWAIA1YSTPYLVDYGSGKANLQSKIFFIWFGCCFLCIAFV
AN1797.1  	LKARAKCLSITTAAN~WLLNWAIA~YATPYMVDSGPGNANLQSKVFFIWGGFCLIAGIFV
          	** **: **:***:* **:***** * ***:*: *.* ******:**:* * *::.  **

MG06203.1 	YFFIYETKGLSLEEVDELYAQVSSARHSSAWQPSVSFTQRTGVDS----ATVEKIEGDAS
NCU04963.1	YFMIYETKGLTLEQVDELYEEVSDARKSIGWVPTITFREIR---------------EEKK
FG02641.1 	YFFIYETKGLSLEEVDQLYDEVSVASKSKQWKPSESWEHRQSVSG----ADGKMFGGNDH
AN1797.1  	WTCIYETKGLTLEEVDELYAKVPVAWRSKGFVPSVHYTDVRDVAAGRKGSGLAELEADAQ
          	:  *******:**:**:** :*. * :*  : *:  : .  .  .. ..:       :  

MG06203.1 	~DHAVDQANRV---
NCU04963.1	0NICVAAALK----
FG02641.1 	~QGEVTHNEKNEA-
AN1797.1  	~AKQISEHVEKASV
          	    :    .  :
```
